# Long term change of the optic disc and OCT parameters during myopic shift in children with large cup to disc ratio

**DOI:** 10.1371/journal.pone.0235621

**Published:** 2020-07-17

**Authors:** Ye Jin Ahn, Yoo Yeon Park, Shin Hae Park, Sun Young Shin

**Affiliations:** Department of Ophthalmology and Visual Science, Seoul St Mary’s Hospital, College of Medicine, The Catholic University of Korea, Seoul, Republic of Korea; Nicolaus Copernicus University, POLAND

## Abstract

**Purpose:**

This observational case series was to determine long term optic disc changes in eyes with large cup to disc ratio (CDR) and compare the changes induced by myopic shift during childhood with normal control eyes.

**Methods:**

Children under 15 years of age who developed myopia with serial optic disc photographs and spectral domain (SD)-optical coherence tomography (OCT) images with a minimal interval of three years were evaluated. Children with average CDR ≥ 0.6 on SD-OCT were classified as having large CDR. The ratios of vertical disc diameter (VDD), horizontal disc diameter (HDD), and maximum peripapillary atrophy (PPA) width (PPW) were measured to quantify morphologic changes of optic discs and SD-OCT parameters, such as peripapillary retinal nerve fiber layer (RNFL) thickness and macular ganglion cell and inner plexiform layer (GCIPL) thickness were measured.

**Results:**

Of the 82 eyes (82 patients) analyzed, 42 eyes had large CDR and 40 eyes were normal controls. The mean age and refractive error at initial examination were not different between groups (P = 0.33, P = 0.76, respectively). The changes in HDD/VDD and PPW/VDD ratios during follow-up showed no significant difference among the groups (P = 0.45, P = 0.62, respectively). No statistical significance was found in changes in RNFL and GCIPL thickness between the two groups (P = 0.74, P = 0.79, respectively).

**Conclusions:**

Children with enlarged CDR showed changes in optic disc morphology and RNFL/GCIPL thickness similar to normal children during myopic shift.

## Introduction

Tilted appearance and temporal crescent are characteristic features of myopic disc which is an acquired condition, since progressive tilting of the optic nerve head (ONH) and development/enlargement of peripapillary atrophy (PPA) take place during childhood myopic shift [1, 2]. These optic disc changes were not only observed in normal children, but also in children with an enlarged cup to disc ratio (CDR) [3].

Introduction of optical coherence tomography (OCT) has enabled measurement of quantitative ONH parameters and retinal nerve fiber layer (RNFL) thickness values and can be performed noninvasively in children [4, 5]. In an effort to establish normative values of RNFL thickness in children, numerous studies have been performed using spectral domain (SD)-OCT, and several groups have reported that RNFL thickness decreased with more negative refractive status [6–9]. Formerly, large discs with large CDR in children were considered physiologic [10, 11]. However, a previous study found 13% of eyes with large CDR progress to definite glaucoma over three years [12], and assuming refraction has a positive effect on RNFL thickness, children with large CDR during myopic shift are more likely to undergo glaucomatous damage.

Some population-based studies have suggested myopia as an associated factor for the development of glaucoma [13–15]. ONH deformations in myopia may predispose toward glaucoma, and the direction of disc tilt may correspond to the location of RNFL loss or visual field defects [16–18]. Unfortunately, whether children with large CDR are at higher risk of developing glaucoma over time is unknown and no study has been performed to determine optic disc changes in this age group.

Therefore, the aim of this study was to compare the morphological changes in the optic disc induced by myopic shift during childhood between eyes with enlarged CDR and normal controls.

## Methods

This observational case series was approved by the institutional review board of Seoul Saint Mary’s Hospital and the study protocol followed the guidelines of the Declaration of Helsinki. Written informed consent forms were obtained from the parents of the children. Patients under 15 years of age who developed myopia with serial optic disc photographs and spectral domain (SD)-OCT at Seoul St. Mary’s Hospital from January 2008 to December 2018 were included in this study.

Children with average CDR ≥ 0.6 on SD-OCT were classified in the large CDR group, and those with CDR < 0.6 were assigned to the normal control group. Exclusion criteria included the following: best-corrected visual acuity (BCVA) worse than 20/25, SE more than -6 diopters (D), intraocular pressure (IOP) ≥ 21 mmHg at initial examination, pathological disc and cup, such as morning glory syndrome, optic nerve hypoplasia, or tilted disc syndrome, abnormal findings on fundus photography, systemic illness, abnormal developmental history, previous trauma or ocular surgery, and non-cooperation from children. Patients were required to have at least two optic disc photographs and two SD-OCT images with an interval of at least 3 years, and those with a time period less than 3 years between the baseline and final examinations were not included in this study.

All patients underwent overall ophthalmic examination, including measurement of BCVA, refractive error, IOP, and axial length (AL) by way of the IOL master (IOL master 500, Carl Zeiss, Jena, Germany), as well as slit-lamp biomicroscopy. Spherical equivalent (SE) was measured under cycloplegic refraction. Optic disc photographs were taken using a nonmydriatic fundus camera (Topcon, Tokyo, Japan). To ensure that the disc shape change was not the result of different photography angle, the final disc photograph was overlaid onto the baseline disc photograph and the blood vessel contour around the optic disc served as a reference. Only photographs with similar angle of viewing were included for analysis. The vertical disc diameter (VDD), horizontal disc diameter (HDD), and maximum peripapillary atrophy (PPA) width (PPW) were measured from the baseline and final optic disc photographs using ImageJ software (available at http://rsb.info.nih.gov/ij/index.html). The ratios of HDD to VDD (HDD/VDD) and PPW to VDD (PPW/VDD) were calculated. For sub-group analysis, patients were classified into eyes with ONH/PPA change and eyes without ONH/PPA change, according to the method described by Kim et al. [2] SD-OCT (Cirrus HD-OCT, Carl Zeiss, Jena, Germany) was performed with FAST RNFL thickness protocols using internal fixation. Data on peripapillary RNFL (RNFL) thickness, macular ganglion cell and inner plexiform layer (GCIPL) thickness, rim area, disc area, average CDR, vertical CDR, and cup volume were obtained and only data with a signal strength greater than 6 without any motion artifacts were used. Parental CDR was also measured, and children with either a father or mother having CDR ≥ 0.6 were considered to have disc suspect parents.

For statistical analysis, all data were analyzed using the SPSS Statistics 19.0 software (IBM Corporation, Armonk, NY, USA). Only one randomly chosen eye was considered for each patient. The comparison between two groups was performed with c*hi*-square and Student *t*-tests. To identify the factors associated with the ONH/PPA change, univariate and multivariate logistic regression analyses were performed. The variables with a significance of P < 0.1 upon univariate analysis were included in the multivariate model. Correlation studies were performed using linear regression. A P value less than 0.05 was considered to be statistically significant.

## Results

114 eyes of 114 patients were examined, however, 32 patients were excluded for having a follow-up period shorter than 3 years. Finally, a total of 82 eyes of 82 patients were included in the present study. The control group consisted of 40 eyes and the large CDR group consisted of 42 eyes. The mean ages of each group were 7.34±3.03 years in the control group and 8.05±3.26 years in the large CDR group (P = 0.33). Patients in the large CDR group had more disc suspect parents with CDR ≥ 0.6 (P = 0.08). The baseline mean SE was -1.15±3.94D in the control group and -0.93±2.15D in the large CDR group (P = 0.76). Myopic shift in the large CDR group (-1.81±1.71D) was greater than the control group (-1.47±2.08D), however, the difference was not statistically significant (P = 0.43). The baseline mean axial length and the axial elongation during follow-up also did not show statistical difference between groups (P = 0.96, P = 0.31, respectively) (Table 1).

**Table 1:** Demographic and clinical characteristics.

	Large CDR group (n = 42)	Control group (n = 40)	P-value
Age (years)	8.05±3.26	7.34±3.03	0.33*
Sex (Male:Female)	23:19	20:20	0.67†
Disc suspect parents	16	5	0.08†
IOP (mmHg)			
Baseline	15.56±2.84	15.12±2.88	0.53*
Final	15.68±3.40	15.07±2.72	0.46*
SE (D)			
Baseline	-0.93±2.15	-1.15±3.94	0.76*
Final	-2.74±2.76	-2.30±3.78	0.56*
Changes	-1.81±1.71	-1.47±2.08	0.43*
Axial length (mm)			
Baseline	23.46±1.17	23.48±1.71	0.96*
Final	24.7351.22	24.12±1.83	0.20*
Changes	0.75±0.71	0.96±0.72	0.31*

CD, cup/disc ratio; IOP, intraocular pressure; SE, spherical equivalent; D, diopter.

*Comparison by Independent t-test.

†Comparison by chi-square test.

Table 2 describes the optic disc and peripapillary tissue characteristics on optic disc photography in the two groups. The HDD/VDD ratio was not different between the groups at baseline (P = 0.46) or at final examination (P = 0.99). The change in the HDD/VDD ratio was also not different between groups (P = 0.45). Likewise, the PPW/VDD ratio showed no difference both at baseline (P = 0.18) and at final examination (P = 0.53), and the change in the PPW/VDD ratio was not significantly different (P = 0.62).

**Table 2:** Optic disc morphological changes on fundus photography in eyes with large CDR and normal controls.

	Large CDR group (n = 42)	Control group (n = 40)	P-value
Follow-up periods (months)	52.55±22.02	51.70±23.25	0.87
Horizontal disc diameter to vertical disc diameter ratio			
Baseline	0.874±0.090	0.893±0.133	0.46
Final	0.862±0.108	0.863±0.128	0.99
Changes	-0.011±0.089	-0.030±0.125	0.45
Maximum peripapillary width to vertical disc diameter ratio			
Baseline	0.067±0.082	0.099±0.127	0.18
Final	0.127±0.119	0.148±0.166	0.53
Changes	0.059±0.096	0.049±0.095	0.62

CDR, cup/disc ratio.

As the previously used time domain (TD)-OCT images are not compatible with SD-OCT images, the mean follow-up periods of ONH parameters with OCT were shorter compared to optic disc photography follow-up periods (control group, 41.10±14.69 months; large CDR group, 44.52±20.36 months; P = 0.21) (Table 3). The average RNFL thickness at baseline and final follow-up were not significantly different between groups (P = 0.44, P = 0.34, respectively), as well as the changes during follow-up (P = 0.74). The changes between baseline and final examinations of the average CDR, vertical CDR, disc area, and cup volume were not statistically significant. On the other hand, the average GCIPL thickness, as well as the six surrounding segments, at baseline, final, and changes between baseline and final examinations, also did not show significant difference.

**Table 3:** Optic disc parameters on optical coherence tomography in eyes with large CDR and normal controls.

	Large CDR group (n = 42)	Control group (n = 40)	P-value
Follow-up periods (months)	44.52±20.36	41.10±14.69	0.21
Peripapillary RNFL			
Average thickness (μm)			
Baseline	95.28±10.25	97.83±17.60	0.44
Final	92.58±10.93	95.44±14.90	0.34
Changes	-1.47±4.77	-1.94±7.38	0.74
Superior thickness (μm)			
Baseline	117.80±18.50	119.00±25.57	0.81
Final	112.05±20.93	115.88±23.46	0.45
Changes	-3.40±7.30	-3.37±14.44	0.99
Inferior thickness (μm)			
Baseline	120.88±16.42	126.56±22.83	0.20
Final	116.29±16.46	121.90±27.00	0.26
Changes	-3.33±10.84	-5.11±9.56	0.45
Temporal thickness (μm)			
Baseline	75.02±12.37	77.79±23.80	0.52
Final	74.90±12.03	76.38±22.11	0.71
Changes	-1.23±10.70	-1.13±9.02	0.97
Nasal thickness (μm)			
Baseline	67.62±9.77	68.05±13.87	0.87
Final	64.17±10.73	62.43±13.51	0.52
Changes	-2.26±8.56	-4.05±8.51	0.36
Rim area (mm^2^)			
Initial	1.16±0.28	1.52±0.45	<0.001
Final	1.24±0.30	1.44±0.36	0.009
Changes	0.06±0.47	-0.0006±0.38	0.53
Disc area (mm^2^)			
Baseline	2.47±0.53	2.24±0.49	0.05
Final	2.37±0.44	2.08±0.47	0.006
Changes	-0.19±0.46	-0.08±0.53	0.35
Average CDR			
Baseline	0.73±0.06	0.51±0.15	<0.001
Final	0.67±0.13	0.52±0.17	<0.001
Changes	-0.09±0.22	0.02±0.11	0.06
Vertical CDR			
Baseline	0.68±0.09	0.49±0.14	<0.001
Final	0.62±0.14	0.48±0.17	<0.001
Changes	-0.09±0.22	0.04±0.18	0.07
Cup volume (mm^3^)			
Baseline	0.51±0.27	0.22±0.13	<0.001
Final	0.49±0.30	0.22±0.14	<0.001
Changes	0.49±0.30	0.22±0.14	0.11
GCIPL (μm)			
Average thickness			
Baseline	82.40±4.17	82.56±5.11	0.86
Final	79.73±8.29	80.70±7.74	0.60
Changes	-0.57±1.75	-0.38±2.96	0.79
Minimal thickness			
Baseline	79.75±4.81	79.30±8.47	0.84
Final	78.81±7.06	76.59±11.97	0.34
Changes	-0.09±2.52	-1.78±11.20	0.49
Superior thickness			
Baseline	82.35±3.77	79.87±17.06	0.53
Final	81.94±7.39	80.97±8.26	0.60
Changes	0.83±1.80	2.79±16.01	0.56
Superotemporal thickness			
Baseline	82.45±4.06	81.61±5.03	0.55
Final	80.73±6.42	79.68±8.15	0.54
Changes	-0.04±3.14	-0.29±2.56	0.77
Inferotemporal thickness			
Baseline	82.65±5.42	82.39±5.13	0.87
Final	79.73±6.82	79.46±9.59	0.89
Changes	-1.57±2.54	-1.29±3.28	0.75
Inferior thickness			
Baseline	80.75±5.27	81.61±6.18	0.63
Final	78.24±7.51	79.67±8.66	0.46
Changes	-170±4.26	0.33±4.72	0.13
Inferonasal thickness			
Baseline	82.30±4.26	82.83±6.25	0.75
Final	81.22±7.28	81.32±8.99	0.96
Changes	-0.61±2.54	-0.21±6.37	0.78
Superonasal thickness			
Baseline	83.55±5.03	84.83±5.33	0.43
Final	82.81±6.61	80.59±14.95	0.41
Changes	-0.09±2.39	-4.13±15.24	0.22

CDR = cup/disc ratio; RNFL = retinal nerve fiber layer; GCIPL = ganglion cell-inner plexiform layer

**Fig 1:**
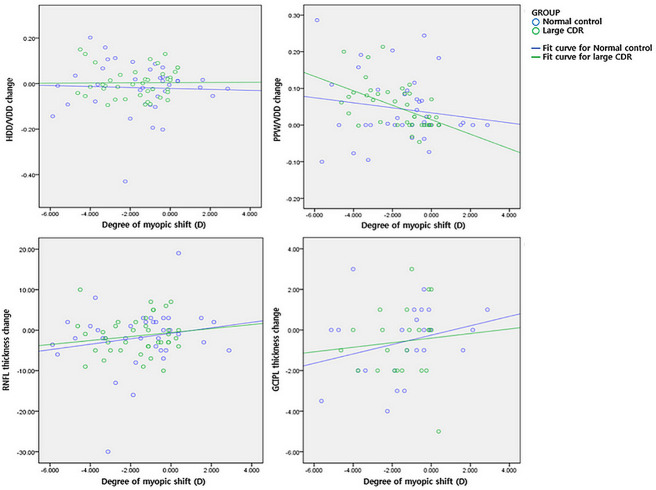
The relationship between degree of myopic shift (diopters, D) and the changes in optic disc morphology, as well as OCT parameters. The horizontal disc diameter (HDD) to vertical disc diameter (VDD) ratio merely changed, while the peripapillary atrophy width (PPW) to vertical disc diameter (VDD) ratio increased as the subjects underwent myopic shift. However, the changes were not statistically significant in either group (upper panel). Both retinal nerve fiber layer (RNFL) and ganglion cell and inner plexiform layer (GCIPL) thickness decreased as the eyes became more myopic, but the changes were not significant in either group (lower panel).

The change in the HDD/VDD ratio (control group; R^2^ = 0.002, P = 0.816, large CDR group; R^2^ = 0.001, P = 0.852), RNFL thickness (control group; R^2^ = 0.036, P = 0.265, large CDR group; R^2^ = 0.030, P = 0.283), and GCIPL thickness (control group; R^2^ = 0.081, P = 0.176, large CDR group; R^2^ = 0.008, P = 0.683) did not show significant correlation with SE change during follow-up. However, the change in PPW/VDD ratio (control group; R^2^ = 0.205, P = 0.034, large CDR group; R^2^ = 0.411, P = 0.029) was significantly correlated with the degree of myopic shift in both groups (Fig 1).

**Fig 2:**
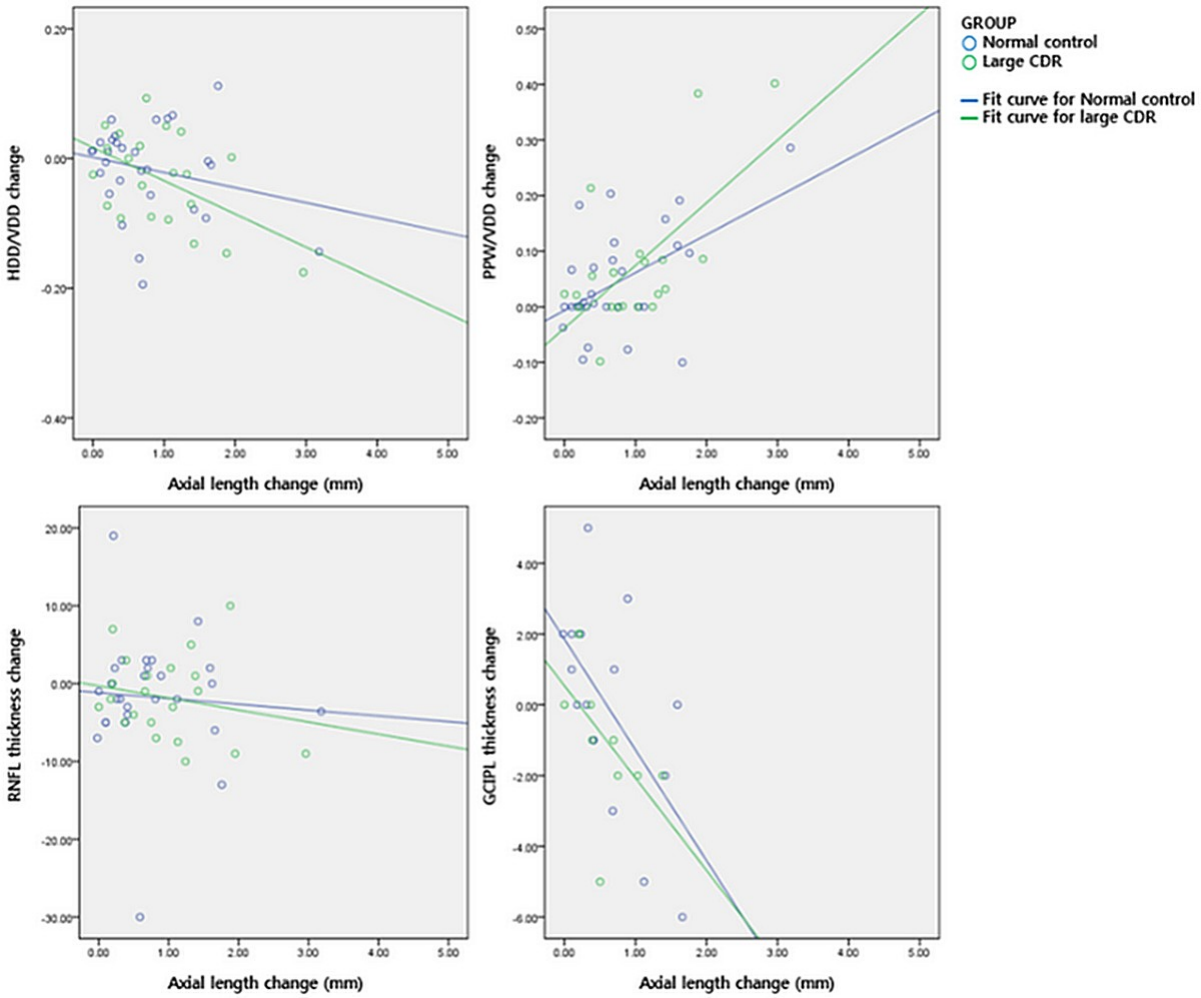
The relationship between the degree of axial length change (mm) and the changes in optic disc morphology, as well as OCT parameters. The horizontal disc diameter (HDD) to vertical disc diameter (VDD) ratio and the peripapillary atrophy width (PPW) to vertical disc diameter (VDD) ratio significantly changed as the subjects underwent axial elongation (upper panel). Retinal nerve fiber layer (RNFL) merely decreased with AL change, but ganglion cell and inner plexiform layer (GCIPL) thickness significantly decreased as the eyes became longer in both groups (lower panel).

**Fig 3:**
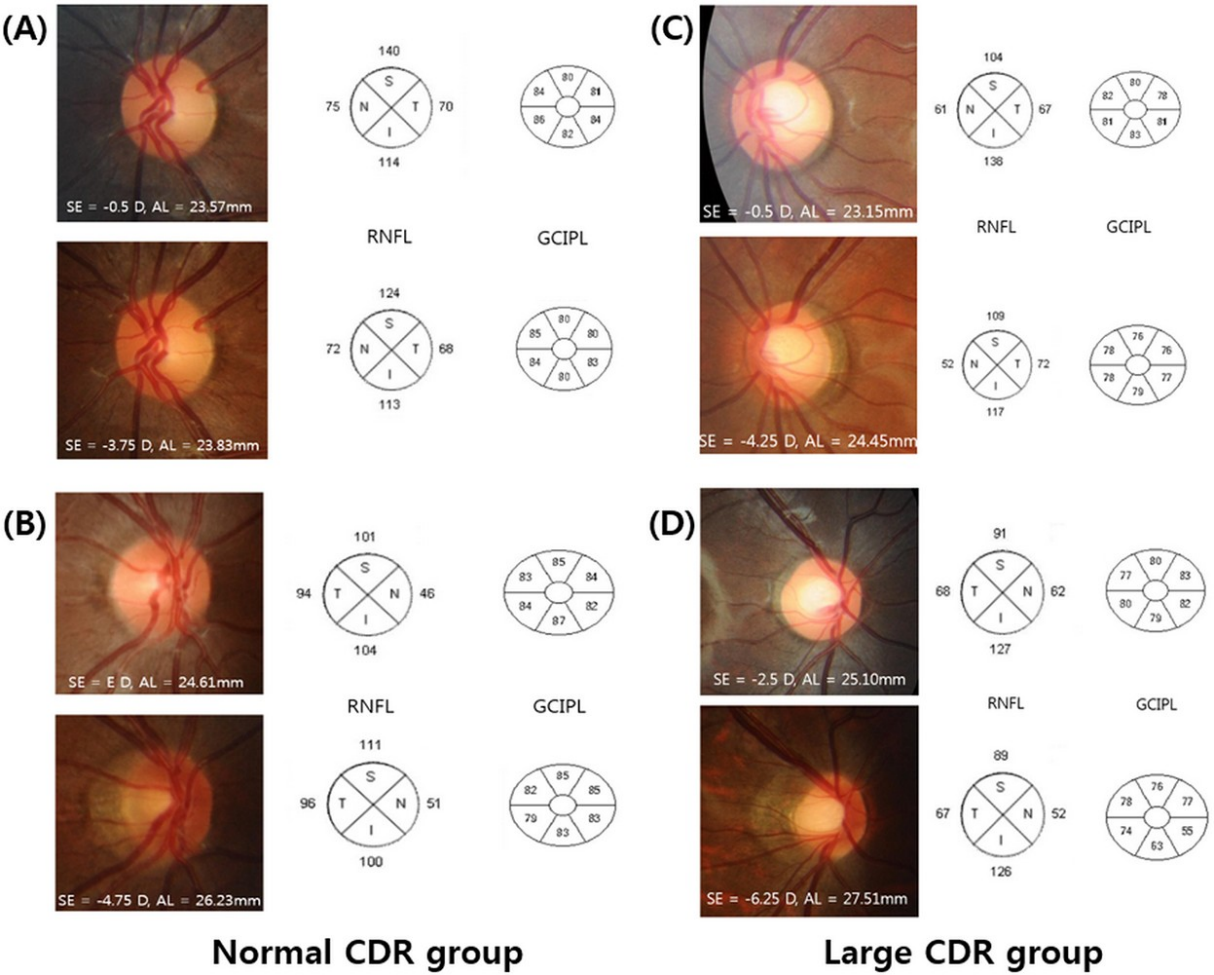
Four cases of childhood eyes with normal optic disc (A and B) and eyes with enlarged CDR (C and D) during myopic shift are presented. The refractive error status (spherical equivalent, SE) and axial length (AL), retinal nerve fiber layer (RNFL) and ganglion cell and inner plexiform layer (GCIPL) thickness at baseline examination are shown in the upper panel, while the parameters at final examination are shown in the lower panel. Eyes with greater AL elongation during follow-up showed more prominent disc tilting and development/enlargement of PPA in both the normal control and large CDR group. However, the RNFL and GCIPL thickness changes were not significant.

Meanwhile, the change in the HDD/VDD ratio (control group; R^2^ = 0.054, P = 0.022, large CDR group; R^2^ = 0.258, P = 0.0.019), PPW/VDD ratio (control group; R^2^ = 0.262, P = 0.0.005, large CDR group; R^2^ = 0.433, P = 0.001), and GCIPL thickness (control group; R^2^ = 0.360, P = 0.014, large CDR group; R^2^ = 0.278, P = 0.012) showed significant correlation with AL change during follow-up. The change in RNFL thickness (control group; R^2^ = 0.005, P = 0.737, large CDR group; R^2^ = 0.043, P = 0.369) did not correlate with axial elongation in either group (Fig 2). The representative cases are shown in Fig 3.

When classified according to the ONH morphology change, 37 eyes were sorted into the ONH/PPA change group and 45 eyes into the ONH/PPA unchanged group. The baseline SE was not different (P = 0.17), but the final SE was more myopic in the ONH/PPA change group compared to the ONH/PPA unchanged group (P = 0.001), and the mean myopic shift was greater in the ONH/PPA change group (-2.50±1.82 D) than the ONH/PPA unchanged group (-0.95±1.66 D, P<0.001). The ONH/PPA change group had longer AL at both baseline (P = 0.03) and final examination (P = 0.001), and the ONH/PPA change group underwent greater axial elongation compared to the ONH/PPA unchanged group (P = 0.008). Age at baseline, number of disc suspect parents, IOP at baseline, distribution of eye with large CDR, and change in mean RNFL and GCIPL thickness did not differ between groups (Table 4).

**Table 4:** Comparison between patients with or without optic disc morphological changes.

	ONH/PPA unchanged group (n = 45)	ONH/PPA change group (n = 37)	P-value
Age (years)	7.30±3.10	8.26±3.19	0.19*
Sex (Male:Female)	26:19	17:20	0.29†
Disc suspect parents	37	24	0.07†
Distribution of groups (large CDR:normal)	19:26	23:14	0.07†
IOP (mmHg)	15.32±3.10	15.38±2.58	0.94*
SE (D)			
Baseline	-0.60±2.81	-1.57±3.45	0.17*
Final	-1.42±3.08	-3.89±3.00	0.001*
Changes	-0.95±1.66	-2.50±1.82	<0.001*
Axial length (mm)			
Baseline	23.14±1.34	23.90±1.54	0.03
Final	23.80±1.53	25.30±1.32	0.001*
Changes	0.61±0.51	1.24±0.85	0.008*
Horizontal disc diameter to vertical disc diameter ratio			
Baseline	0.87±0.70	0.90±0.15	0.40*
Final	0.90±0.10	0.82±0.12	0.001*
Changes	0.03±0.09	-0.08±0.10	<0.001*
Maximum peripapillary width to vertical disc diameter ratio			
Baseline	0.06±0.10	0.11±0.11	0.06*
Final	0.07±0.11	0.21±0.15	<0.001*
Changes	0.01±0.05	0.11±0.11	<0.001*
Average CDR	0.61±0.17	0.64±0.14	0.34*
Average RNFL thickness (㎛)			
Baseline	97.80±15.85	95.03±12.15	0.40*
Final	94.28±12.81	93.57±13.27	0.81*
Changes	-2.78±6.49	-0.43±5.54	0.09*
Average GCIPL thickness (㎛)			
Baseline	82.96±4.72	81.94±4.61	0.49*
Final	80.58±9.96	79.79±5.05	0.66*
Changes	-0.04±2.79	-1.05±1.70	0.13*

ONH = optic nerve head; PPA = peripapillary atrophy; IOP = intraocular pressure; SE = spherical equivalent; D = diopter; CDR = cup/disc ratio; RNFL = retinal nerve fiber layer; GCIPL = ganglion cell-inner plexiform layer.

* Comparison by Independent t-test.

† Comparison by chi-square test.

Logistic regression analysis was performed to identify which factors are related to the ONH/PPA change (Table 5). Based on univariate analysis, whether the subject had disc suspect parents or not, change in SE, baseline AL, and change in AL were factors related to the change in ONH morphology. On multivariate analysis, the baseline AL and the AL change turned out to be the final related factors to the ONH/PPA change.

**Table 5:** Related factors to optic disc morphological changes in patients with or without large CDR.

	Univariate analysis			Multivariate analysis		
	OR	95% CI	p-value	OR	95% CI	p-value
Age	1.104	0.954–1.277	0.185			
Sex (M)	1.610	0.670–3.867	0.287			
Disc suspect parents	0.399	0.144–1.107	0.077	0.313	0.119–1.099	0.141
IOP	1.006	0.851–1.190	0.941			
Average CDR	0.470	0.228–1.922	0.341			
Baseline SE	0.901	0.777–1.045	0.168			
SE change	0.592	0.437–0.802	0.001	0.579	0.396–0.848	0.058
Baseline AL	1.466	1.023–2.101	0.037	1.627	0.986–2.685	0.047
AL change	2.479	1.496–3.414	0.007	0.612	0.436–0.860	0.013

CDR = cup/disc ratio; OR = odds ratio; CI = confidence intervals; M = male; IOP = intraocular pressure; SE = spherical equivalent; AL = axial length

## Discussion

We compared changes in the optic disc morphology and OCT parameters that occur with myopia during childhood under normal and enlarged CDR conditions. Only disc suspect children were evaluated and those with childhood glaucoma were not included in the present study. The mean age, SE, and axial length were not different between groups at baseline examination and showed similar changes throughout the follow-up period. The changes in optic disc ovality and the development of or increase in PPA during childhood myopic shift, as well as changes in RNFL and GCIPL thickness, did not show statistical difference between the two groups.

Scleral stretching associated with the axial elongation during myopic shift has been suggested for the cause of progressive disc tilting and development/enlargement of PPA in myopic eyes [2]. Another study by Park et al [3] demonstrated that optic disc changes during myopic shift can differ among various conditions and showed greater changes in disc suspects while childhood glaucoma eyes showed significantly less changes. As corneal hysteresis has been reported to be reduced in eyes with glaucoma [19], the authors hypothesized that altered biomechanics of the ocular tissue may have resulted in different responses of the ONH complex and sclera to axial elongation.

In the current study, the HDD/VDD ratio and PPW/VDD ratio, which represents optic disc morphology, were not correlated with the degree of myopic shift during the follow-up period in both the normal control and the large CDR group. On the other hand, HDD/VDD ratio and PPW/VDD ratio showed significant correlation with AL change during follow-up. The large CDR group underwent greater change than the normal control group, however the difference was statistically insignificant. Regression analysis also revealed ONH/PPA change was associated with baseline AL and AL change, but not with SE change nor baseline CDR. As shown in the representative cases, more pronounced disc shape changes were seen in eyes with greater AL elongation, even with similar myopic shift in refraction (Fig 3). These changes were observed both in the normal control group and the large CDR group. Our findings may represent that morphologic changes of the optic disc are mostly affected by AL change. Other ocular biometries, such as corneal power, anterior chamber depth, lens thickness, and vitreous chamber depth were not measured. Moreover, three-dimensional visualization of the posterior pole has been introduced using the En face program, which can reconstruct the eyeball in the anteroposterior orientation, enabling the evaluation of the deepest point of eyeball [20]. Further investigation on these parameters might give explanation of the discrepancy between AL and SE change in this study.

OCT has become a widely used tool in clinical ophthalmology, and a number of studies investigating normative data for children have shown that RNFL thickness decreases with less positive refractive error [6, 8, 21] and increasing axial length [22–24]. Tsai et al. reported that the average RNFL thickness increased by 1.7 μm for every diopter change towards hyperopia [21]. Recently, some authors observed that macular GCIPL thickness in normal children positively correlated with SE [25, 26] and negatively correlated with AL [25, 27, 28]. However, in the present study, we evaluated the OCT parameter changes over 36 months, and the thickness of the nerve fiber layer and the ganglion cell layer were not correlated with SE change. On the other hand, GCIPL thickness decreased with AL elongation, but RNFL thickness showed no correlation with AL change; these results were demonstrated regardless of CDR. Parikh et al. claimed that RNFL losses happen later in life, after the age of 50 years [29]. Our results also suggest that although optic discs change as myopia progresses, RNFL thickness does not change during childhood regardless of whether the CDR is enlarged. Compared to the RNFL thickness, which was measured in the peripapillary area, the macular GCIPL thickness was more greatly affected by AL change, as the posterior pole region is more vulnerable to axial elongation than the peri-optic nerve region [30].

Our study has several limitations that must be acknowledged. First, it had a relatively small sample size and it is difficult to generalize our findings because all of the subjects were referred to a tertiary hospital and only Korean patients were included. The findings might be unique to this population, in which myopia prevalence and progression rate is high, and may not be applicable to other populations. Second, visual field test was not performed because it is difficult to perform and is not routinely carried out with children. However, damage to the RNFL precedes visual field loss, and up to 40–50% of the RNFL could be lost before visual field defects are detected [31]. Thus, RNFL measurements may be used as a surrogate marker for visual field defects.

In conclusion, during myopic shift, optic disc tilting and development/enlargement of PPA, as well as changes in the RNFL and GCIPL thickness, were not different between eyes with large CDR and normal controls. Morphologic optic disc changes and OCT parameter changes were affected by the amount of AL change, irrelevant of CDR.
